# Quantifying Long-Term Adaptations in Performance Variables in Adolescent Athletes: A 1.5 Year Longitudinal Training Study Utilising a Standardised, Progressive, Blocked Linear Periodisation Resistance Training Program

**DOI:** 10.3390/sports13060164

**Published:** 2025-05-27

**Authors:** Michael A. Carron, Vincent J. Dalbo

**Affiliations:** 1 School of Health, Medical and Applied Sciences, Central Queensland University, Rockhampton 4702, Australia; m.carron@cqu.edu.au; 2 Health, Education, Lifestyle, and Performance (HELP) Laboratory, St Brendan’s College, Yeppoon 4703, Australia

**Keywords:** youth, strength training, strength and conditioning, gym, fitness

## Abstract

We examined the effects of resistance training over 1.5 years (two seasons). Body mass, strength, power, and aerobic capacity were assessed during the first 1.5 years of a standardised, progressive, blocked linear periodisation training program in adolescent males with no prior history of resistance training (N = 11, 16.4 ± 0.5 years). Testing occurred during the start of pre-season (SPS), end of pre-season (EPS), and end of season (EOS) during the first and second year of resistance training. Changes over time were assessed with within-group ANOVAs and follow-up independent *t*-tests. Differences in relative change that occurred during the first and second year of training for each variable were assessed with dependent *t*-tests. Body mass changed over time (*p* < 0.001, *n^2^p* = 0.794). Body mass increased from SPS to EPS (*p* = 0.008, *Large*) and EPS to EOS (*p* = 0.019, *Large*) in year 1, and from EOS in year 1 to SPS in year 2 (*p* < 0.001, *Large*). Bench press (*p* < 0.001, *n^2^p* = 0.806), squat (*p* < 0.001, *n^2^p* = 0.670), and medicine ball throw (*p* < 0.001, *n^2^p* = 0.350) changed over time. Bench press (Year 1: *p* < 0.001, *Large*; Year 2: *p* < 0.001, *Large*), squat (Year 1: *p* < 0.001, *Large*; Year 2: *p* < 0.001, *Large*), and medicine ball throw (Year 1: *p* = 0.007, *Large*; Year 2: *p* = 0.026, *Large*) increased from SPS to EPS in year 1 and year 2. Bench press (*p* = 0.010, *Large*) and squat (*p* = 0.004, *Large*) increased more from SPS to EPS during year 1 than year 2. By quantifying diminished returns, our study offers practitioners novel information, helping practitioners manage expectations, avoid excessive training and support long-term adolescent development.

## 1. Introduction

As recently as the early 1970s, gym-based training was believed to hinder on-field player performance. This view began to change when Boyd Epley was hired as the first full-time strength and conditioning coach at the University of Nebraska in 1969 and was able to help improve the performance of their football team through gym-based exercise [1]. Since that time, there has been great interest in the ability of resistance training to improve the health and performance of adolescent (<18 years), college-age (18–35 years), middle-aged (>35–64 years) and older individuals (≥65 years). Indeed, the positive effects of resistance training are so pervasive that most adolescents who partake in sport during high school engage in a strength and conditioning program to reduce their injury risk and enhance their performance [2]. In an attempt to develop an optimal strength and conditioning program, practitioners are encouraged to develop an annual plan (i.e., macrocycle) that is composed of smaller blocks of training, termed mesocycles (i.e., a cycle of training that may last weeks to months), which are composed of smaller blocks of training, termed microcycles (i.e., the structure of training during the hypertrophy cycle of training; it can be structured in weekly or daily blocks).

Despite the belief that structured, progressive resistance training programs will yield optimal performance outcomes, most published research has quantified the effects of resistance training on muscle strength and other performance outcomes over a single phase or mesocycle [3,4,5,6,7]. As such, most resistance training programs are conducted over periods of twelve weeks or less [3,4,5,6,7] and are designed to represent a single mesocycle. However, a well-established negative correlation exists between training experience and the rate of progression, whereby the more trained an individual becomes, the more slowly their performance improves [8]. The reduced rate of progression with enhanced training experience is the result of diminished physiological adaptations occurring as an individual moves closer to their genetic potential [9], and this phenomenon has been termed the ceiling effect. The ceiling effect is widely accepted, but we are only aware of a few studies that have specifically sought to quantify the decreased rate of progression that occurs following chronic resistance training ≥1 year [8,10,11,12]. Importantly, the studies that we are aware of [8,10,11,12] were conducted retrospectively, and only one study [10] employed a standardised resistance training protocol. As a result, research is needed to quantify the effects of standardised, progressive resistance training protocols on performance outcomes to establish expected rates of progression among adolescent athletes longitudinally. Furthermore, research is needed to quantify the degree of diminishing returns among adolescent athletes to fill the gap concerning the expected longitudinal returns from periodisation programming, to assist coaches in optimising workloads, managing expectations, and supporting long-term athlete development. Consequently, we quantified the physiological adaptations that occur in adolescents with no prior history of resistance training over their first 1.5 years of following a standardised, progressive, linear periodisation program designed for adolescent rugby league athlete development.

## 2. Methods

A longitudinal experimental design was utilized to monitor physiological adaptions that occur in response to a standardised, progressive, blocked linear periodisation training program occurring over two seasons. Data were collected over a period of 1.5 years (i.e., March 2023 to September 2024) in adolescent, male athletes with no previous history of resistance training. Outcome variables for the study were as follows: body mass, one-repetition maximum (1RM), barbell bench press (bench press), 1RM barbell back squat (squat), medicine ball throw (MBT), counter-movement jump (CMJ) and multi-stage fitness test (MSFT).

### 2.1. Participants

All participants were recruited from the same schoolboy rugby league team prior to the commencement of the 2023 season, and their training and performance was monitored from February 2023 to September 2024, which corresponded with the start of training for the 2023 rugby league season and the conclusion of the 2024 rugby league season. To be included in the study, participants had to have no prior history of resistance training, had to qualify for the first-grade rugby league team during the 2023 season and had to be in grade 11 or 12. Athletes were excluded from the study if they were in grade 12 (i.e., seniors) as it would not have been possible to monitor their performance during the following season due to graduation.

The study started with 25 potential participants (e.g., boys who qualified for the first-grade rugby team and volunteered to participate in the study) but ended with 11 participants. Potential participants were excluded due to having prior resistance training experience (*n* = 1), not being selected to the first-grade rugby team during the 2023 season (*n* = 2) or being a senior during the 2023 season (*n* = 6). Of the 16 participants meeting the inclusion criteria, 5 participants were lost due to incomplete data from testing occasions (i.e., did not complete more than one entire testing session, including gym- and field-based tests). As a result, 11 participants were observed across the 1.5-year testing period (age: 16.4 ± 0.5 years; age range: 16–17 years). Notably, 4 participants concurrently partook in an external rugby league academy program, which exposed them to additional gym-based and field-based training. Written assent was obtained from each participant, and written consent was obtained from the legal guardian of each participant prior to the commencement of the study. All study procedures were approved by the Human Research Ethics Committee (#0000023904).

### 2.2. On-Field Training Program

Throughout each macrocycle, on-field training occurred on Monday and Wednesday from the start of the pre-season to the end of the season. Each Monday session consisted of an additional 15 min video analysis and team meeting session, prior to on-field training. Each on-field training session typically consisted of conditioning (i.e., bouts of sprinting over 10-, 20- and 40-m distances with rest intervals ranging from 15 to 60 s), conditioning drills including small-sided games in conjunction with bouts of high-intensity interval training (i.e., running) or coach-led field training consisting of skill performance (e.g., tackle simulation, passing and kicking), drills (e.g., set plays), game-based conditioning (i.e., aerobic capacity) and opposed sessions (i.e., practice/friendly games). On-field training programs were revised for individual athletes if an injury was present. Each on-field session typically lasted 45 min in duration in the pre-season and 30 min while in season, and no on-field training sessions occurred during the off-season.

### 2.3. Resistance Training Program

Weekly gym-based training was composed of an upper-body lift on Monday, a lower-body lift on Wednesday and a whole-body lift on Friday. Each gym-based exercise session started with a 15 min warm-up. The warm-up consisted of light aerobic cycling activity (5 min), various mobility exercises targeting major functional movements with dynamic stretches targeting the lower and upper-body (4 min), movement-preparatory exercises with body weight or resistance bands (e.g., banded squats, lunges, chest presses and rows, 4 min) and passing a football repeatedly across 10 m intervals while moving at different intensities (2 min).

The upper-body lift consisted of the selection of a primary exercise followed by four supplementary exercises and one trunk exercise. The lower-body lift consisted of the selection of a primary exercise followed by four supplementary exercises and one trunk exercise. The whole-body lift consisted of two primary exercises (i.e., one upper-body primary exercise and one lower-body primary exercise) followed by four supplementary exercises (i.e., two upper-body supplementary exercises and two lower-body supplementary exercises) and one trunk exercise. Table 1 presents the list of primary and supplementary exercises that were utilized for upper-body, lower-body, and whole-body exercises. The resistance training program followed the principles of a traditional linear blocked periodization program (i.e., macrocycle) with a progression in load of 3% to 5% when applicable (i.e., when athletes were able to complete the prescribed sets, repetitions and load). When power was an aim of the mesocycle (e.g., during pre-season), one supplementary exercise was removed from the training program and three plyometric exercises for the upper-body or lower body were included in the training program depending on the focus of the specific training session (e.g., if the upper-body was being trained, the three plyometric exercises selected would be upper-body plyometric exercises). During the power phase of the mesocycle for whole-body days, one upper (all performed bilaterally) and two lower plyometric exercises were selected (i.e., one bilateral and one unilateral exercise).

### 2.4. Two-Year Macrocycle

**Figure 1:**
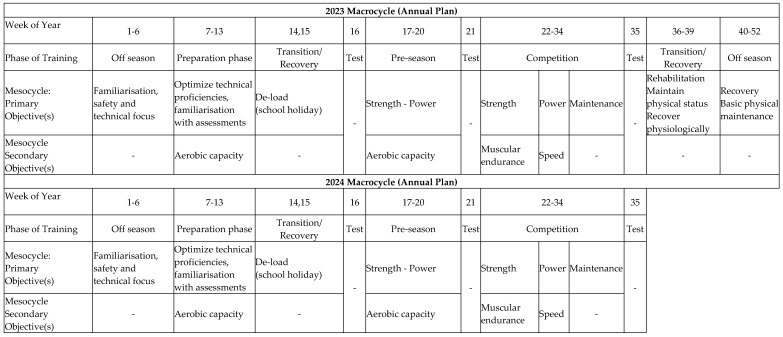
The 1.5 year (two-season) linear blocked periodisation program.

Our 1.5 year resistance training program was composed of two one-year-long macrocycles focused on preparing the participants to compete in a first-grade rugby league competition. Each year-long macrocycle employed a gradual increase in training intensity with a decrease in training volume over the course of the seasonal phases, while utilising a blocked strategy consisting of mesocycles with an emphasis on improving specific physical qualities (i.e., strength and power) to support rugby league performance. Our standardised, progressive, linear, blocked periodisation program (Figure 1) was composed of 10 mesocycles across the two-season training period (preparation phase, pre-season phase, competition phase, transition phase/recovery phase and off-season phase; these 5 phases occurred during each year of training). Each of the 5 repeated mesocycles was broken down into microcycles, which were also repeated during each year of training.

### 2.5. Off-Season, Preparation and Transition Phases of Training

The start of each calendar year commenced with participants in the off-season (weeks 1–6), in which no on-field or resistance training was prescribed. During weeks 7–13, participants engaged in the preparation phase of training, which was composed of two on-field sessions per week and three resistance training sessions per week, which were broken down into upper-body training on Monday, lower-body training on Wednesday and full-body training on Friday. Resistance training sessions lasted 45 min and were focused on teaching proper technique and developing skeletal muscle as a result. A self-selected load was used in this study to prioritise technical competency and ensure safety among untrained individuals during the preparatory phase of training, given that untrained participants often are not competent enough to perform strength assessments (e.g., 1RM testing) to derive data or possess the motor control required for maximal loading. Therefore, allowing participants to self-select their load during the preparatory phase of training enabled participants to work at an intensity appropriate to their ability while minimising the injury risk. Participants were required to perform warm-up sets with the bar before adding a self-determined amount of load under guided instruction, placing an emphasis on technique. Participants were informed to cease performing the exercise when they believed that they could only complete 1–2 more repetitions with the load. Moreover, athletes were familiarised with all testing protocols. Weeks 14–15 served as a transition/recovery phase as the athletes were away for school holidays, and no on-field or resistance training sessions were prescribed during this time.

### 2.6. Start of Pre-Season Testing and Pre-Season Phase

Week 16 served as baseline testing, so no on-field or resistance training sessions were scheduled during this week, and participants were instructed to refrain from vigorous activity for 48 h prior to testing, which occurred on Wednesday and Friday. Weeks 17–20 served as the pre-season phase which was composed of two on-field sessions per week and three resistance training sessions per week. The resistance training sessions were broken down into upper-body training on Monday, lower-body training on Wednesday, and full-body training on Friday. Resistance training sessions lasted 45 min and were focused on strength. As a result, 5 sets were typically performed per lift at 85% 1RM for primary exercises and 3 sets were typically performed per lift at 85% 1RM for secondary exercises.

### 2.7. End of Pre-Season Testing

Week 21 served as follow-up testing (end-pre-season), so no on-field or resistance training sessions were scheduled during this week, and participants were instructed to refrain from vigorous activity for 48 h prior to testing, which occurred on Wednesday and Friday.

### 2.8. In-Season Phase

Weeks 22–34 served as the in-season phase, which was composed of two on-field sessions per week and two resistance training sessions per week, which were broken down into upper-body training on Monday and full-body training on Friday, except for weeks containing a game, which comprised of full-body training on Monday and Friday. Resistance training sessions for weeks 22–26 lasted 30 min and were focused on maintaining skeletal muscle and strength for the initial four-week mesocycle; as a result, five sets were typically performed per lift at 85% 1RM for primary exercises, and three sets were typically performed per lift at 85% 1RM for secondary exercises. Resistance training sessions lasted 30 min and were focused on power for the second four-week mesocycle for weeks 26–30. As a result, three sets were typically performed per lift at 80% 1RM for primary exercises, and three sets were typically performed per lift at 75% 1RM for secondary exercises. For plyometric-based activities, athletes were instructed to perform movements at 80–100% maximal violation[3]. Resistance training sessions for weeks 30–34 lasted 30 min and were focused on maintaining performance (maintenance); they consisted of one team-based rehabilitation session and one strength session to maintain skeletal muscle. As a result, five sets were typically performed per lift at 85% 1RM for primary exercises, and three sets were typically performed at 85% 1RM for secondary exercises.

### 2.9. End-of-Season Testing and Off-Season

Week 35 served as end-of-season testing; no on-field or resistance training sessions were scheduled during this week, and participants were instructed to refrain from vigorous activity for 48 h prior to testing, which occurred on Wednesday and Friday. Weeks 36–40 served as a transition/recovery period as the athletes were away for school holidays, and no on-field or resistance training sessions were prescribed during this time. The end of each calendar year concluded with participants in the off-season (weeks 40–52), in which no on-field or resistance training was prescribed.

### 2.10. Testing Protocol for Outcome Variables

The testing of performance outcomes comprised six tests, implemented during the pre-season, end of pre-season and end-of-season phases of training. The administration of the six tests was conducted in accordance with an established methodology [13]. All tests included in the protocol have been reported to be reliable [14] and can be utilized to monitor potential changes over time [15]. Athletes were instructed to refrain from vigorous activity, consume their typical diets and undertake their normal daily schedules (i.e., sleep, hydration and school participation) for 48 h prior to each testing session [16]. Prior to the first testing session of each season, all participants were familiarized with each test throughout the duration of the preparation phase (i.e., weeks 7–13), in which participants received verbal instruction, visual demonstrations and practice attempts on all tests on at least two occasions. Participants were only allowed to partake in the 1RM lifts after gaining clearance from the strength and conditioning coach, who held a Master’s degree in strength and conditioning. For all tests, verbal encouragement was provided by the experimenter, and athletes were tested during the same time of day.

### 2.11. Body Mass

Body mass was measured using electronic scales (SECA 813, SECA Corp) with athletes wearing normal rugby league attire (i.e., shirt and shorts), without footwear or socks. The body mass assessment protocol utilized has been reported to be reliable in adolescents (coefficient of variation (CV) = 0.63%; interclass correlation coefficient (ICC) = 0.999) [14].

### 2.12. One-Repetition-Maximum Barbell Bench Press

Maximal upper-body strength was assessed using the 1RM barbell bench press. Testing was performed in accordance with the procedures outline by the National Strength and Conditioning Association (NSCA) [17]. Specifically, a 1RM bench press had to be obtained in a maximum of five attempts, and a minimum of 180 s of seated rest was provided between attempts [17]. All attempts were completed using a 2.13 m Olympic bar (20 kg). The bench press was assessed with hands pronated using a hook grip and athletes maintaining a supine position on a flat bench. The 1RM barbell bench press assessment protocol utilized has been reported to be reliable in adolescents (CV = 1.08%, ICC = 0.988) [14].

### 2.13. One-Repetition-Maximum Barbell Back Squat

Maximal lower-body strength was assessed using the 1RM barbell back squat. Testing was performed in accordance with the procedures outline by the NSCA [17]. Specifically, the 1RM squat had to be obtained in a maximum of five attempts, and a minimum of 180 s of seated rest was provided between attempts [17]. All attempts were completed using a 2.13 m Olympic bar (20 kg). The back squat was performed with self-selected footing using a high bar position and a pronated hook grip. For a squat attempt to be counted as successful, athletes had to reach a depth where their thighs were parallel to the floor. The 1RM barbell back squat assessment protocol utilized has been reported to be reliable in adolescents (CV = 1.12%, ICC = 0.985) [14].

### 2.14. Medicine Ball Throw

Maximal upper-body power was assessed with the MBT. The MBT required participants to throw a 2 kg medicine ball (Celsius; Australia) horizontally to achieve the maximum distance (m) possible. Athletes were seated (side saddle) on a flat bench placed flush against a wall. Throughout the duration of the MBT attempt, athletes were required to have their scapula (i.e., shoulder blades) in contact with the wall, remain seated with knees flexed to 90° and have their feet in full contact with the floor. Participants were required to grip the medicine ball in both hands, bring the medicine ball to their chest and push the medicine ball away from their chest with both hands, throwing the medicine ball as far as possible. A measuring tape was fastened to the floor at the wall positioned directly below the athlete when sitting on the bench, with the distance measured in a direct line from the wall to the initial floor marking (indicating the landing of the ball, which was covered in chalk) to the nearest 1 cm. The experimenter positioned the measuring tape in line with the closest point of contact from the ball to the participant. The MBT assessment protocol utilized has been reported to be reliable in adolescents (CV = 3.72%, ICC 0.733) [14].

### 2.15. Counter-Movement Jump

The CMJ required participants to stand erect with both arms fully extended overhead; while in this position, the experimenter adjusted the height of the yardstick device (Swift; Lismore, Australia) to ensure that the first vane was at fingertips reach for each participant. In one fluid motion, athletes squatted to a depth of their choice while swinging their arms below their waist and jumped vertically as high as possible utilizing an arm swing. To obtain the vertical jump height, athletes were required to displace a vane on the yardstick device. Athletes completed three attempts, with 60 s of passive standing rest between attempts. For all attempts, the CMJ height was measured to the nearest 1 cm, with the best attempt recorded for analysis. The CMJ assessment protocol utilized has been reported to be reliable in adolescents (CV = 4.02%, and ICC = 0.874) [14].

### 2.16. Multi-Stage Fitness Test

Aerobic capacity was estimated in one attempt using the MSFT, following established procedures [18]. The MSFT involves repeated 20-m shuttle runs with progressive increases in speed dictated by audio cues. The MSFT was concluded when participants either voluntarily stopped or failed to cover the requisite distance as signalled by audio cues across two successive shuttles. Maximal oxygen uptake (VO2max [mL/kg/min]) was estimated from the last successful shuttle completed during the MSFT using a valid equation [18]. The MSFT assessment protocol utilized has been reported to be reliable in adolescents (CV = 3.04% and ICC = 0.840) [14].

## 3. Statistical Analysis

Normality and homogeneity of variance for each outcome variable were confirmed using the Shapiro–Wilk (*p* > 0.05) and Levene’s tests (*p* > 0.05), respectively (Jamovi software; version 2.3.28; the Jamovi project). As a result, parametric statistics were utilized for each statistical analysis, and data are reported as the mean ± standard deviation (SD). Prior to data collection, it was determined that each statistical analysis would be conducted to test for repeatability, with *a priori* alpha level of <0.05 needing to be met for a finding to be deemed significant; meaningfulness was determined utilizing an appropriate effect size statistic. As the significance and meaningfulness of a finding may provide conflicting results, it was determined prior to data collection that all statistical decisions (e.g., whether to break down a model such as a within-group ANOVA with separate dependent *t*-tests) were made based on significance. The collected data are presented in two phases to provide a comprehensive analysis of the performance adaptations that occurred in response to the 1.5 year-long resistance training protocol.

During phase one, separate within-group ANOVAs (6 timepoints) were performed for each dependent variable to analyse the effects of the 1.5-year resistance training protocol on each dependent variable over time. The effect size of each within-group ANOVA was quantified using the partial eta-squared (*n^2^p*), with the effect size magnitudes interpreted as follows: *small* = 0.01, *moderate* = 0.06 or *large* = 0.14 [19]. If a significant effect was found to occur for a within-group ANOVA, follow-up dependent *t*-tests were conducted. To reduce the probability of making a type I error, the follow-up dependent tests compared each timepoint to the successive timepoint (i.e., timepoint 1 was compared to timepoint 2, timepoint 2 was compared to timepoint 3, etc.). A standardised Hedge’s *g* (g) was utilized as the effect size to quantify whether meaningful differences occurred between timepoints due to the small sample size [20] and was reported with 95% confidence intervals (95% CI). The magnitude of the effect size of Hedges’ *g* was determined as *trivial*: <0.20, *small*: 0.20–0.49, *medium*: 0.50–0.79 or *large*: ≥0.80; however, if the 95% CI overlapped with 0, the magnitude of the effect was defined as *trivial* regardless of the effect size. The Jamovi software (version 2.3.28; the Jamovi project) was used to analyse the within-group ANOVAs, *n^2^p* and subsequent dependent *t*-tests. The standardised Hedge’s *g* and the respective 95% CIs were quantified manually using Microsoft Excel (version 15, Microsoft Corp; Redmond, WA, USA) as follows.

Step 1: Quantify Standardised Cohen’s d [20].

Cohen’s *d* = (Timepoint A Mean–Timepoint B Mean)/Pooled Standard Deviation.

We utilized (Post-Test Timepoint–Pre-Test Timepoint)/Pooled Standard Deviation.

Step 2: Quantify Standardised Hedges’ *g* [20].

Hedges’ *g* = d*(1-(3/(4*(Number of Pairs-1)-1))),

where d = Cohen’s d.

Step 3: Quantify 95% Confidence Interval for Standardised Hedges’ *g*.

95% CI for Hedges’ *g* = g ± (t_α/2_*SE_g_),

where *g* = the effect size of the standardised Hedges’ *g*;

t_α/2_ = the critical value from a dependent-samples t-distribution table [21]*;

SE_g_ = the standard error of Hedges’ *g* quantified as

SE_g_ = √((1/Number of Pairs) + (g^2/(2*Number of Pairs)))).

*Note*: The t-critical value is predicated on the number of paired samples and was adjusted to account for missing data when applicable. * Where n = 11, df = n − 1 and t-critical = t_2.201_; where n = 10, df = n − 1 and t-critical = t_2.228_ [21].

During phase two, we converted the raw values utilized in phase one of the study into change values to quantify potential differences in relative change that occurred in the first year of training compared to the second year of training. Differences in relative change were quantified as subsequent timepoint-previous timepoint. As a result, we have two timepoints for year 1 of training and two timepoints for year 2 of training. Timepoint 1 for each year of training was quantified as: end of pre-season-start of pre-season. Timepoint 2 for each year was quantified as end of season-end of pre-season. After quantifying the differences in relative change for each year and timepoint, separate dependent *t*-tests were used to detect potential repeatable differences that occurred in the relative change for each outcome variable in participants during their first year of training compared to their second year of training (Jamovi software, version 2.3.28; the Jamovi project). The standardised Hedges’ *g* (*g*) with 95% CIs was utilized as the effect size measure to quantify potential differences between timepoints (i.e., end of pre-season–start of pre-season year 1–end of pre-season–start of pre-season year 2)/pooled standard deviation)). The standardised Hedges’ *g* and the 95% CIs were quantified manually using Microsoft Excel (Microsoft Excel, version 15, Microsoft Corp; Redmond, WA, USA).

## 4. Results

### 4.1. Phase One: Athlete Progression over the First 1.5 Years of Resistance Training

Results from phase one of the study are presented in Table 2. The results from the within-group ANOVA revealed a significant, *large* change in body mass over time (*p* < 0.001, *n^2^p* = 0.794). Follow-up dependent *t*-tests revealed a significant, *large* increase in body mass from the start of pre-season to the end of pre-season during the first year of training (*p* = 0.008, *g* = 0.91, 95% CI = 0.11 to 1.71); a significant, *medium* increase from the end of pre-season and end of season in the first year of training (*p* = 0.019, *g* = 0.77, 95% CI = 0.01 to 0.54); and a significant, *large* increase from the end of season in the first year of training to the start of pre-season in the second year of training (*p* < 0.001, *g* = 2.37, 95% CI = 1.03 to 3.71).

Results from the within-group ANOVA revealed a significant, *large* change in the 1RM bench press over time (*p* < 0.001, *n^2^p* = 0.806). Follow-up dependent *t*-tests revealed a significant, *large* increase in the 1RM bench press from the start of pre-season to the end of pre-season during the first year of training (*p* < 0.001, *g* = 1.72, 95% CI = 0.65 to 2.80) and second year of training (*p* < 0.001, *g* = 1.85, 95% CI = 0.72 to 2.97).

Results from the within-group ANOVA revealed a significant, *large* change in the 1RM back squat over time (*p* < 0.001, *n^2^p* = 0.670). Follow-up dependent *t*-tests revealed a significant, *large* increase in the 1RM squat from the start of pre-season to the end of pre-season during the first year of training (*p* < 0.001, *g* = 1.92, 95% CI = 0.77 to 3.08) and second year of training (*p* < 0.001, *g* = 1.38, 95% CI = 0.43 to 2.33).

Results from the within-group ANOVA revealed a significant, *large* change in the MBT over time (*p* = <0.001, *n^2^p* = 0.350). Follow-up dependent *t*-tests revealed a significant, *large* increase in MBT from the start of pre-season to the end of pre-season during the first year of training (*p* = 0.007, *g* = 0.94, 95% CI = 0.14 to 1.77) and a significant, *trivial* increase in the second year of training (*p* = 0.026, *g* = 0.73, 95% CI = −0.03 to 1.48).

Results from the within-group ANOVA revealed a non-significant, *moderate* change in the CMJ over time (*p* < 0.279, *n^2^p* = 0.115). Results from the within-group ANOVA revealed a non-significant, *moderate* change in the MSFT over time (*p* = 0.825, *n^2^p* = 0.173). 

### 4.2. Phase Two: Comparison of the Relative Change That Occurs During the First and Second Year of Resistance Training

**Figure 2:**
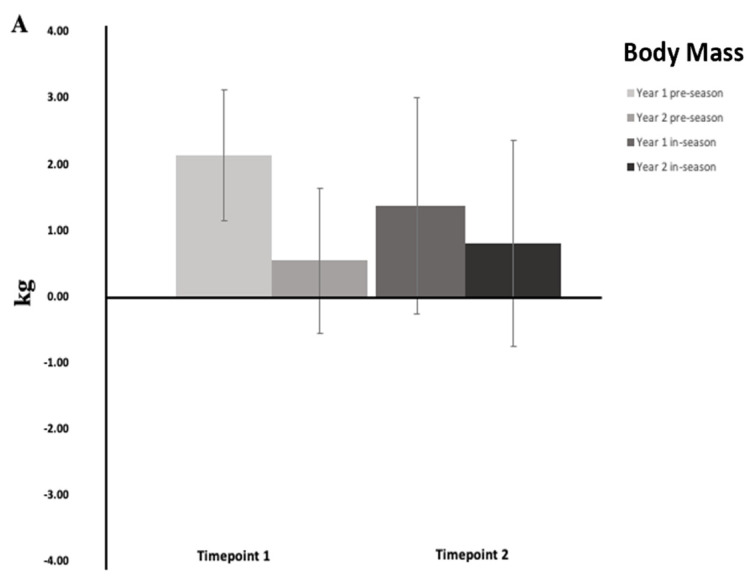

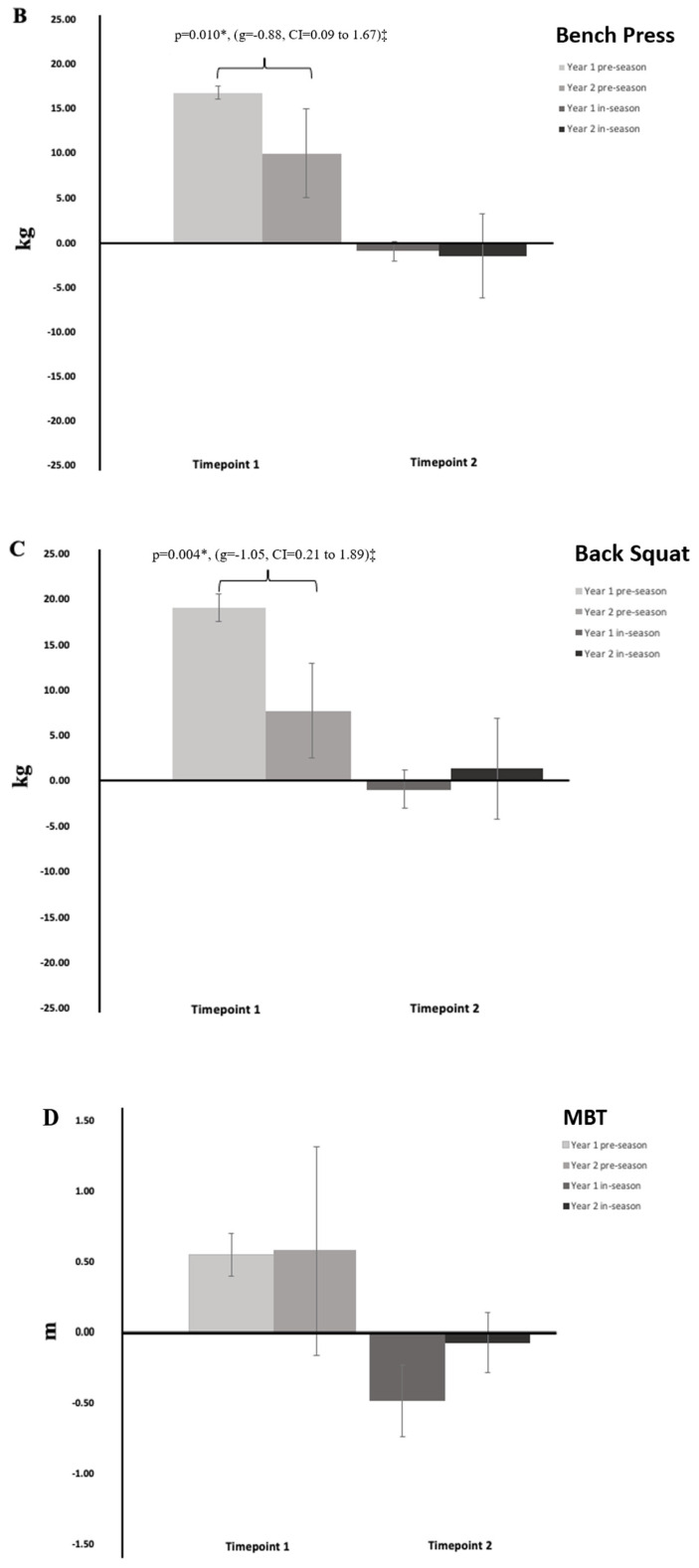

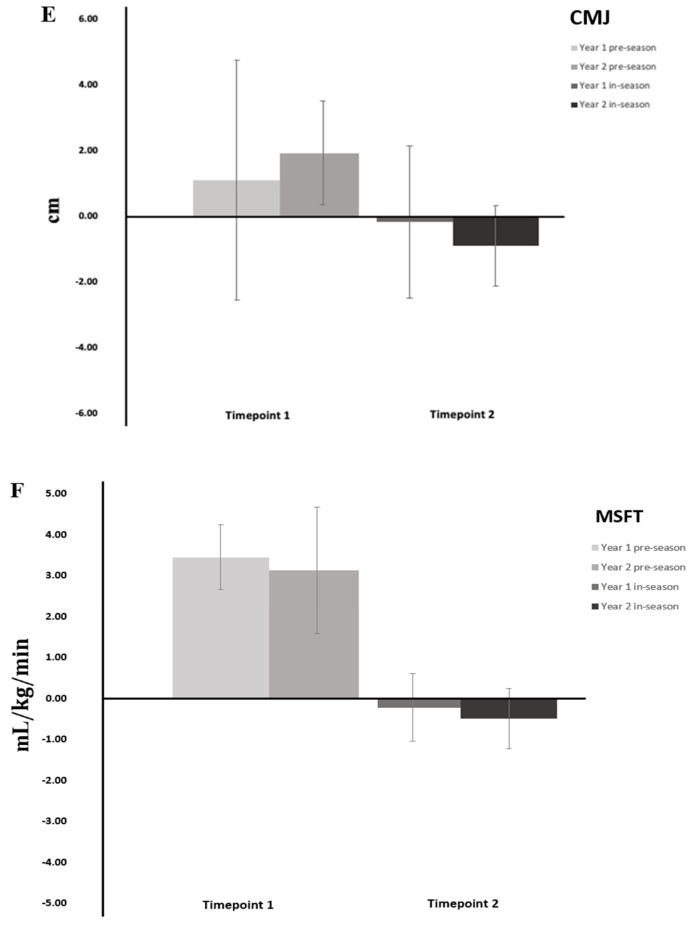
Differences in relative change that occurred during timepoint 1 (change from start of pre-season to start of in-season) and timepoint 2 (change from start of in-season to end of season) during the first and second year of resistance training. Notes: (**A**) Body mass (kg); (**B**) 1RM bench press (kg); (**C**) 1RM back squat (kg); (**D**) medicine ball throw (m); (**E**) counter-movement jump (cm); (**F**) multi-stage fitness test (mL/kg/min). * denotes a significant difference between timepoints determined by separate dependent *t*-tests. ‡ denotes a meaningful difference between timepoints (effect size magnitude ≥ small).

A comparison of the relative change that occurred in each outcome variable across the pre-season and in-season phases of training during the first and second year of resistance training is presented in Figure 2. A non-significant, *trivial* effect was revealed for body mass at timepoint 1 (*p* = 0.088, *g* = −0.53, 95% CI = −1.24 to 0.19) and timepoint 2 (*p* = 0.314, *g* = −0.30, 95% CI = −0.97 to 0.38). A significant, *large* difference was revealed for the 1RM bench press at timepoint 1 as participants experienced a larger increase in strength during their first year of resistance training (*p* = 0.010, *g* = −0.88, 95% CI = −1.67 to −0.09), while a non-significant, *trivial* difference was revealed between years of training at timepoint 2 (*p* = 0.64, *g* = −0.13, 95% CI = −0.84 to 0.58). A significant, *large* difference was revealed for the 1RM back squat at timepoint 1 as individuals experienced a larger increase in strength during their first year of resistance training (*p* = 0.004, *g* = −1.05, 95% CI = −1.89 to −0.21), while a non-significant, *trivial* difference was revealed between years of training at timepoint 2 (*p* = 0.461, *g* = 0.21, 95% CI =−0.46 to 0.89). A non-significant, *trivial* effect was found for the MBT at timepoint 1 (*p* = 0.88, *g* = 0.04, 95% CI = −0.62 to 0.71) and timepoint 2 (*p* = 0.12, *g* = 0.47, 95% CI = −0.23 to 1.18). A non-significant, *trivial* effect was found for the CMJ at timepoint 1 (*p* = 0.66, *g* = 0.13, 95% CI = −0.54 to 0.79) and timepoint 2 (*p* = 0.57, *g* = −0.17, 95% CI = −0.83 to 0.50). A non-significant, *trivial* effect was found for the MSFT at timepoint 1 (*p* = 0.71, *g* = −0.11, 95% CI = −0.77 to 0.56) and timepoint 2 (*p* = 0.76, *g* = −0.09, 95% CI = −0.75 to 0.58).

## 5. Discussion

Nearly all studies examining the effects resistance training on physiological adaptations occur over training durations of ≤12 weeks [3,4,5,6,7] and are typically structured to assess adaptations that occur over a single phase or mesocycle of training. Consequently, there is a paucity of research examining adaptations that occur in response to resistance training over durations of >12 weeks, and even less research exists that examines adaptations that occur in response to resistance training over multiple phases or mesocycles of training. As a result, we quantified the effects of the first 1.5 years of a standardised, progressive, blocked, linear periodisation resistance training program in adolescents with no prior history of resistance training. We quantified the overall effects of the 1.5 year standardised, progressive, blocked, linear periodisation resistance training program on each outcome variable over time with the use of separate within-group one-way ANOVAs, and we quantified the differences in relative change that occurred in each outcome variable during the first year and second year of resistance training with separate dependent *t*-tests to directly assess the ceiling effect. We found significant, large increases to occur in body mass at each timepoint for the first 12 months of training (year 1 start of pre-season to year 2 start of pre-season). Significant, large increases in bench press, squat and MBT occurred from the start of pre-season to end of pre-season during the first and second years of resistance training, with relative change during the first year of training being significantly larger than in the second year of training for bench press and back squat, but with a non-significant, trivial difference occurring for the relative change in the CMJ.

### 5.1. Body Mass

Participants experienced a significant, *large* increase in body mass over the 1.5 year study (baseline: 75.6 ± 8.7 kg; end of study: 84.4 ± 6.9 kg). Participants experienced significant, large increases in body mass from the start of pre-season to the end of pre-season, from the end of pre-season to end of season and from the end of season to start of pre-season the following year, representing the first 12 months of training. At which point, body mass remained statistically unchanged from the start of pre-season in year 2 to the end of season in year 2, representing the final 6 months of training. The large increase in body mass during the first 12 months of training (start of pre-season year 1 to start of pre-season year 2) followed by the stabilization of body mass in the subsequent six months of training (start of pre-season year 2 to end of season year 2), despite participants following a standardised, progressive, blocked, linear periodisation resistance training program, suggests that skeletal muscle accumulation is a meaningful contributor to the increases in body mass and strength that occur upon the onset of resistance training. Our hypothesis is consistent with research demonstrating molecular pathways regulating skeletal muscle growth are activated during the first bout of resistance exercise [22,23,24]. Further, Defreitas et al. [25] reported a significant increase in skeletal muscle cross-sectional area of the midpoint of the thigh between the greater trochanter and lateral epicondyle to occur in previously untrained college-age males following two resistance training sessions, assessed with a peripheral quantitate computed tomographic (pQCT) imaging device. The authors [25] acknowledged muscular edema may have contributed to the initial finding of increased muscle cross-sectional area after two resistance training sessions but were confident muscle cross-sectional area significantly increased following three to four weeks of resistance training (~9–12 resistance training sessions), as the increase in muscle cross-sectional area after 3–4 weeks of resistance training corresponded with the first significant increase in isometric maximal voluntary contraction, which occurred in week 4. We also found the largest performance increases to occur in bench press and back squat during the start of pre-season to the end of pre-season in year one, which is strongly suggestive of the accretion of skeletal muscle mass and neural adaptations.

Our findings regarding changes in body mass are in agreement with the work of Waldron et al. [26], who reported body mass to significantly increase in adolescent rugby league athletes (age: 15–17 years) assessed at the pre-season across three seasons (year 1: 81.9 ± 9.1 kg; year 2: 86.1 ± 6.0 kg, year 3: 86.3 ± 9.4 kg). Till et al. [27] also reported younger (under 14 years of age) rugby league athletes to experience a greater percent change in body mass when assessed pre- and post-season (7.4 ± 4.3%), compared to older rugby league athletes (under 16 s: 5.2 ± 5.0%; under 18 s: 2.5 ± 4.7%; under 20 s: 1.2 ± 3.3%). While Till et al. [27] did not speculate as to why the diminished increases in body mass occurred with advancing age, their findings may have been the result of younger age groups being less trained and thus more responsive to resistance training stimuli. Furthermore, given the sample population was in the developmental phase of puberty, we recognise that, in part, some of the observed changes in body mass may be attributed to maturation.

### 5.2. Maximal Muscular Strength

Participants experienced a significant, *large* increase in muscular strength across our 1.5-year training study, as indicated by improvements in the 1RM bench press (baseline: 81.8 ± 11.9 kg; end of study: 106.0 ± 9.9 kg) and 1RM back squat (baseline: 104.1 ± 16.9 kg, end of study: 129.5 ± 10.8 kg). The largest improvements in the 1RM bench press and 1RM back squat occurred from the start of pre-season to the end of pre-season during the first and second year of resistance training, with the largest increases in strength occurring during the start of pre-season to end of pre-season in the first year of training. Moreover, neuromuscular efficiency likely increased [28], and individuals would have become more biomechanically efficient at performing lifts over time [29], with greater improvements likely to be experienced during the early phases of training/learning. The emphasis of the resistance training program from the start of pre-season to the end of pre-season was also structured to emphasise strength adaptations, and is consistent with reported findings of a previous systematic review and meta-analysis [30].

Maximal upper-body (i.e., bench press) and lower-body (i.e., back squat) strength was maintained during the season (i.e., end of pre-season to the end of season) during each year of training. We expected this result as the in-season phase of training was designed to maintain performance gains achieved during the pre-season phase of training, as the focus of training shifted to focus on on-field performance. Our finding of strength being maintained during the in-season phase of training in agreement with other research conducted in adolescent rugby league athletes [15,30].

Bench press and back squat 1RM remained unchanged from the end of season in year 1 to the start of pre-season in year 2. This could have been due to several factors. Firstly, at the start of the study participants were 16.4 ± 0.5 years of age. As a result, during the off-season, the loss of training gains could have been offset by the gains in strength incurred from maturation. Secondly, athletes were not instructed to perform resistance training during the off-season, but they were not precluded from strength training. Thus, some participants may have improved their strength by resistance training in the off-season, which could have offset potential decrements in strength experienced by participants who did not resistance train during the off-season. Thirdly, the start of pre-season testing occurred during week 16 of the season; as a result, all participants were exposed to seven weeks of resistance training prior to the strength testing in year 2. Testing occurred in this manner to ensure participants’ safety, but it is possible participants regressed during the off-season and regained their lost strength during the initial preparation phase of training that occurred prior to the start of pre-season testing.

Results from our study clearly support evidence of the ceiling effect. Specifically, we found improvements in 1RM bench press and 1RM back squat strength to significantly and meaningfully slow from the first year to the second year of training, as assessed by the relative change from the start of pre-season to end of pre-season. Specifically, improvements in 1RM bench press strength during the second year of training were −0.88 (95% CI: −1.67 to −0.09) standard deviations below the improvements made during the first year of training. The effects were even more pronounced in 1RM back squat, as improvements made during the second year of training were −1.05 (95% CI: −1.89 to −0.21) standard deviations below the improvements made during the first year of training. Although research examining chronic adaptations is limited, Baker et al.[10] reported younger sub-elite (19.0 ± 0.6 years) male rugby league athletes to experience larger increases in strength, assessed via 1RM bench press, when compared to older elite (21.3 ± 1.4 years) male rugby league athletes. Furthermore, Appleby et al. [12] examined changes in strength among adult rugby union athletes (24.4 ± 3.4 years) across two years. The authors [12] reported 1RM bench press and back squat strength performances increased by 6.5% and 11.5%, respectively, following 2 years of training. Moreover, Appleby and colleagues [12] reported that the magnitude of improvements was negatively associated with initial strength data, meaning individuals who started the study with higher levels of strength experienced lower increases in strength over the course of the study. However, testing only occurred during the pre-season period, and the authors did not account for the seasonal fluctuations in training programs and changes in game-based demands, which influence test performance outcomes. Crucially, our study is the first to quantify the rate of diminishing returns in adolescent athletes over an extended 1.5-year resistance training program. To the best of our knowledge, previous studies have typically examined short timeframes (<1 year) [3,4,5,6,7], making our investigation a valuable contribution to the current understanding of long-term athletic development. Our study provides a practical framework that practitioners may apply to monitor and assess training responses in their own athletes. Measuring the rate of diminishing returns relative to an athlete’s training status (e.g., training age) offers valuable insights into the effectiveness of a given program. As athletes become more trained, performance improvements naturally slow, and, by identifying this early, coaches can avoid unnecessarily increasing training volume in the pursuit of unrealistic improvements. By quantifying the rate of diminishing returns, our findings may better inform practitioners regarding program design and could have positive implications for long-term player development.

### 5.3. Maximal Muscular Power

Participants experienced a significant, *large* increase in upper-body muscular power across our 1.5 year training study, as indicated by improvements in the MBT (baseline: 6.7 ± 0.8 m; end of study: 7.2 ± 0.8 m). The increases in upper-body muscular power may be attributed to concomitant increases in upper-body muscular strength (i.e., 1RM bench press) given significant increases 1RM bench press and MBT each occurred from the start of pre-season to end of pre-season during the first and second year of training. The significant and meaningful improvements in upper-body muscular power and strength corresponded with increases in body mass during the first year of training. Accordingly, we posit a significant and meaningful increase in body mass was primarily composed of increases in lean body mass, given corresponding increases in muscle strength (i.e., upper and lower-body) and upper-body muscular power occurred at this time. Our findings corroborate the findings of Baker [11], who reported a 22% improvement in upper-body muscle power assessed via bench press throw to occur over a 10 year period of time in professional rugby league athletes (N = 6, age at start of study: 19.3 ± 1.6 years), with progression slowing over time.

In contrast, participants experienced a non-significant, *moderate* increase in CMJ performance (baseline: 59.5 ± 6.6 cm; end of season: 61.0 ± 3.5 cm) across our 1.5 year training study. A non-significant finding for lower-body muscular power is surprising, but body mass of the participants increased from 75.6 ± 8.7 kg at the start of the study to 84.4 ± 6.9 kg by the end of the study, meaning participants had to vertically displace significantly greater body mass during successive testing sessions, particularly when compared to the first testing session. CMJ is frequently used to assess lower-body power in research [13], but further consideration may be required when using the CMJ to assess lower-body power in participants with differing body masses or for studies in which the body mass of participants is expected to significantly and/or meaningfully change over time, as improvements in absolute lower-body power can be masked by increases in body weight when assessing lower-body power via the CMJ height. Accordingly, the CMJ is a suitable measure of relative lower-body power, but absolute lower-body power is a valuable measure for practitioners (particularly those who coach athletes in non-weight-dependent sports, such as rugby league) and researchers.

## 6. Limitations

When possible, we recommend the use of a larger sample size, as the predictability of statistical tests improve and reduced likelihood of making a type II error [31]. Specifically, *p*-values become lower and 95% CIs become tighter for the same test outcomes with a larger compared to a smaller sample size. However, longitudinal study designs present challenges, evident by the paucity of research examining training adaptations that occur in interventions of >12 weeks. Further, many studies of longer durations that have examined training adaptations do not report enough information about the training protocol for the study to be replicated. The periodisation and progression of our training program can be replicated in future practice and research, but the exact sessional program implemented (i.e., session exercise selection) cannot be replicated as the exercises performed for each session are not provided, which is a limitation of the present study. Moreover, we acknowledge load was prescribed at a percentage of the 1RM, and the explicit load (kg) was not prescribed or reported for each participant, precluding an examination of total load performed. Indeed, this would require participants to log a diary of exercises performed, loads and the completion of repetitions and sets for the entire duration of the study; we recommend future research consider these limitations in the study designs. In this regard, to build upon our findings, researchers should consider the use of training diaries to enable the comparison of total load prescribed versus total load lifted, which may help researchers to better understand the effects of load on physiological adaptations. Despite our best efforts, we recognise four participants in our study were concurrently enrolled in an external rugby league academy, which exposed them to additional on-field and off-field training over the 1.5 year training period. Participants were also instructed to refrain from lifting during the off-season, but some athletes were likely to have resistance trained during this period of time.

## 7. Conclusions

We quantified physiological adaptations that occurred in adolescents with no prior history of resistance training over their first 1.5 years of following a standardised, progressive, blocked, linear periodisation program designed for adolescent rugby league athlete development. We found the majority of adaptation in body mass to occur during the first 12 months of training. Moreover, increases in body mass corresponded with an increase in 1RM bench press, back squat and MBT; therefore, we posit the body mass adaptation was the result of the accretion of lean body mass, demonstrating the highly responsive nature of the body to adapt to novel training stimuli. Additionally, our findings demonstrate the degree at which returns diminish, given improvements in 1RM bench press and squat were −0.88 and −1.05 standard deviations lower from the start of pre-season to end of pre-season in the second year of training compared to the first year of training.

Generally, the current understanding of the physiological response to resistance training is based on results obtained from acute (≤12 weeks) training studies designed to mimic a single phase or mesocycle of training. However, very few studies report chronic adaptions that occur in response to standardised, progressive, blocked, linear periodisation programs, despite evidence that physiological adaptation in response to training decreases over time. We found that untrained adolescents yielded positive physiological adaptations in response to a standardised, progressive, blocked, linear periodisation program across multiple seasons, with the rates of progression slowing over time. It is broadly acknowledged that rates of progression slow over time, and future research is needed to quantify the expected rate of progression to determine whether training programs yield results that are superior or inferior to the expected rates of improvement given the training status of participants in the study or members of the team. Moreover, researchers should examine how advanced training statuses influence the expression of pathways regulating skeletal muscle growth so that training programs to optimize human development can be based on science rather than intuition.

Key points
  1. Diminished returns occur in adolescents (16 ± 0.5 years) following a standardised, progressive, blocked linear periodisation resistance training program within their first 1.5 years of training.
  2. The majority of physiological adaptations in body mass (84%) and strength (1RM bench press: 68%, back squat: 65%) occurred during the first 12 months of training.
  3. Improvements in strength adaptations were lower by −0.88 (95% CI = −1.67 to −0.09) and −1.05 (95% CI = −1.89 to −0.21) standard deviations for the 1RM bench press and back squat, respectively, from the start of pre-season to end of pre-season in the second year of training compared to the first year of training.

## Figures and Tables

**Table 1:** Primary and secondary exercises used throughout the 1.5 year (two-season) resistance training program.

	*Uppe*r-*Body*			*Lower-Body*			*Full-Body*	*Plyometrics*		*Trunk*
Classification	Overhead	Anterior	Posterior	Anterior	Posterior	Hip/Glute		Upper-Body	Lower-Body	
*Primary*										
	Push Jerk	BB Bench Press	BB Bent Over Rows	Front Squat	Trap Bar Deadlift	Hip Thrust	Clean			
	DB Push Press	BB Incline Bench Press	Seated Row	Back Squat	Romanian Deadlift		Power Clean			
	BB Push Press	BB Decline Bench Press	DB Rows	Overhead Squat			Hang Clean			
	BB Overhead						Full Clean			
***Secondary***										
	Seated DB Overhead Press	DB Bench	Chest Supported Rows		SL Deadlift	Hip Hinge	Clean Pull	Clap Push-up	Alternating Bounds	Sit-ups
	Standing DB Overhead	Incline Bench	Landmine row	Box Squat	DB Deadlift	Back Elevated Hip Up	Clean Shrug	Med Ball Chest Pass	Tuck Jumps	Barbell Rollouts
	DB Cuban Press	Incline DB Bench	BB Inverted Row	Overhead Squat	Band Deadlift	SL Hip Ups	DB Cleans	MB OH Forward Pass		Copenhagen
	Neutral Grip DB Overhead	Dips	Band Rows	Goblet Squat	Below Knee Rack Pull	Band Hip Extensions	KB Cleans	Wall Ball	Broad Jumps	Hanging Leg Raises
	Y-raise, W-raise, T-raise	Band Bench	Straight Arm Pulldown	Hack squat	Above Knee Rack Pull	Hip Adduction	Famers Walk	Med Ball Rotational Throws	Depth Drops	Cable Chop
	Side Delt Raises	Push-ups	Reverse Fly	High Box Squat	SL DB Romanian Deadlift	Hip Abduction		Med Ball Ground Slam	Depth Jumps w/ Bound	Band Rotation Fend
	Rear Delt Raises	Elevated Push-ups	Pullups	Deep Box Squat	DB Romanian Deadlift	Hip Flexion		Lying Medball Chest Pass	Hurdle Hops	
	Face Pulls	Foot Elevated Push-ups	Lat Pulldowns	Band Squat	Reverse Hypers				Lateral Bounds	Planks
	Internal and external Shoulder Rotations	DB Flies	Band Pulldowns	Body weight Squat	Landmine Romanian Deadlift				SL Box Jumps	3 Way Plank
		DB Incline Flies	Chin-ups	5 second Pause Squat	Band Leg Curls				Lateral Bounds	Russian Twists
		Band Push-ups	Inverted Row		Nordic Hamstring Curls				Seated Jump	V-shape Sit-ups
		DB Push-ups			Band Good Morning				Vertical Jumps	Toes to Bar
					Sled Pull				Calf Hops	SA Farmer Walk
					Sled Push				Split Squat Jump	
					BB Lunges				MB Rotational Throws	
					Walking DB Lunges				KB Swings	
					Walking BB Lunges				SL Vertical Jump	
					SL Box Squat				Split Squat Jumps	
					SL Squat					
					SL Hip Lift				SL Hurdle Jumps	
					Lateral Lunges					
					DB Split Squat					
					DB Step Up					
					BB Step Up					
					Power Step Up					
					Leg Extension					

Notes: One primary lift was selected for a session subject to the objective of the session (i.e., one lower-body primary lift was selected on lower-body training days), followed by four secondary lifts and one trunk exercise. For example, on a lower-body day, one may select a back squat from the primary list, followed by a single leg RDL, DB split squat, Nordics, and sled push from the secondary list, followed by barbell rollouts from the trunk list. For full-body days, in general, one upper-body and one lower-body exercise would be selected from the list of primary exercises and two upper-body and two lower-body exercises would be selected from the list of secondary exercises. One trunk exercise was included for upper-body, lower-body, and full-body days. Where the emphasis was on power development, three additional plyometric movements were included during each session. Abbreviations: BB, barbell; DB, dumbbell; RDL, Romanian deadlift; KD, kettlebell; SA, single arm; SL, single leg; MB, medicine ball.

**Table 2:** Means and standard deviations of changes in physical qualities that occurred across the 1.5 year resistance training program.

Test	N	Pre-Season	End Pre-Season	End Season	Pre-Season	End Pre-Season	End Season	Sig (ANOVA)	Partial ETA-Squared (*n^2^p)*
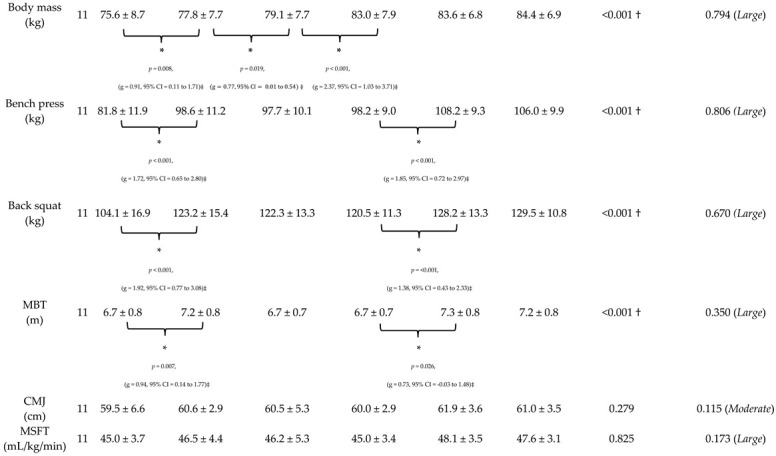									

Notes: † denotes a significant effect for the within-group ANOVA (*p* < 0.05). * denotes a significant difference between timepoints determined by separate dependent *t*-tests (*p* < 0.05). ‡ denotes a meaningful difference between timepoints (effect size magnitude ≥ small).

## Data Availability

Data supporting the findings for this study are available upon reasonable request from the corresponding author.
